# PD‐1/PD‐L1 Inhibitors Plus Chemotherapy Versus Chemotherapy Alone as First‐Line Therapy for Patients With Unfavorable Cancer of Unknown Primary: A Multicenter, Retrospective Cohort Study

**DOI:** 10.1002/mco2.70124

**Published:** 2025-03-06

**Authors:** Riqing Huang, Haifeng Li, Shuo Li, Ditian Shu, Rishang Chen, Zhousan Zheng, Tinghua Gao, Meiting Chen, Anqi Hu, Yunjie Huang, Qiufan Zheng, Xin An, Cong Xue, Yuchen Cai, Yanxia Shi

**Affiliations:** ^1^ State Key Laboratory of Oncology in South China Guangdong Provincial Clinical Research Center for Cancer Sun Yat‐sen University Cancer Center Guangzhou P. R. China; ^2^ Department of Medical Oncology Sun Yat‐sen University Cancer Center Guangzhou P. R. China; ^3^ Department of Pathology Sun Yat‐sen University Cancer Center Guangzhou P. R. China; ^4^ Medical Oncology Department III Central Hospital of Guangdong Nongken Zhanjiang P. R. China; ^5^ Department of Oncology The First Affiliated Hospital of Sun Yat‐sen University Guangzhou P. R. China; ^6^ Department of Oncology The First People's Hospital of Zhaoqing City Zhaoqing P. R. China

**Keywords:** cancer of unknown primary, chemotherapy, first‐line therapy, overall survival, PD‐1/PD‐L1 inhibitors

## Abstract

This multicenter study aimed to investigate the efficacy and safety of PD‐1/PD‐L1 inhibitors plus chemotherapy (ICI‐Chemo group) versus chemotherapy alone (Chemo group) for patients with cancer of unknown primary (CUP) in the first‐line setting. We included patients with unfavorable CUP across four medical centers in China. Between January 2014 and December 2023, 117 patients were enrolled: 46 patients in the ICI‐Chemo group and 71 patients in the Chemo group. After a median follow‐up of 28.1 months, the ICI‐Chemo group exhibited a significant improvement over the Chemo group in median PFS (9.10 months vs. 6.37 months; hazard ratio [HR] 0.46; 95% CI: 0.30–0.71; *p* < 0.001) and OS (35.67 months vs. 10.2 months; HR 0.37; 95% CI: 0.22–0.64; *p* < 0.001). Similarly, among patients who received TP (taxane plus platinum)‐based chemotherapies, OS and PFS benefits were observed in the ICI‐Chemo group. The objective response rate was higher in the ICI‐Chemo group than in the Chemo group (54.35% vs. 22.53%, *p* < 0.001). Grade 3 or higher drug‐related adverse events occurred in 11 patients (23.91%) in the ICI‐Chemo group and 28 patients (39.44%) in the Chemo group. Thus, PD‐1/PD‐L1 inhibitors plus chemotherapy could be the preferred first‐line treatment for patients with unfavorable CUP, providing improved efficacy and manageable toxicity.

## Graphical abstract

We assessed the efficacy and safety of PD‐1/PD‐L1 inhibitors plus chemotherapy versus chemotherapy alone for patients with cancer of unknown primary (CUP) in the first‐line setting. In this cohort study of 117 patients, PD‐1/PD‐L1 inhibitors plus chemotherapy delivered progression‐free survival and overall survival advantages, along with a higher objective response rate, when compared to chemotherapy. The findings indicated that PD‐1/PD‐L1 inhibitors plus chemotherapy could be the preferred first‐line approach for patients with unfavorable CUP.

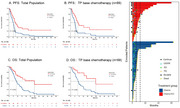

## 1 Introduction

Cancer of unknown primary (CUP) represents a heterogeneous and aggressive malignancy defined by histologically confirmed metastases without an identifiable primary tumor origin responsible for the metastatic spread [1]. CUP accounts for 2%–5% of all diagnosed cancers, with a global incidence rate ranging from 5.3 to 19 cases per 100,000 individuals [2]. According to clinical and histological features, CUP can be stratified into favorable and unfavorable subsets [1]. Traditionally, the initial treatment for patients with unfavorable CUP has involved platinum‐based chemotherapy regimens. However, this strategy offers limited improvement in patient survival, with a median overall survival (OS) of merely 6–13 months [3, 4, 5, 6, 7, 8, 9, 10, 11] and a 1‐year survival rate of 35.6% (95% confidence interval [CI]: 32.0–39.3) [6]. In the clinical setting, patients with unfavorable CUP subsets may be treated with site‐specific chemotherapy based on histologic characteristics or patterns of metastatic spread. Nevertheless, a prior study revealed that the median OS of site‐specific chemotherapy and the empirical treatment (carboplatin plus paclitaxel) was 10.0 months and 10.1 months, respectively, with no significant difference [12]. Additionally, a single‐center randomized trial (Fudan CUP‐001) found that using the 90‐gene expression assay to guide site‐specific therapy improved progression‐free survival (PFS) in patients with previously untreated CUP compared to empirical chemotherapy; however, no significant difference in OS was observed [13]. Given the heterogeneity and aggressiveness of CUP, especially in unfavorable subtypes, it becomes imperative to explore treatment modalities that could prolong survival and improve the prognosis for this patient population.

In recent years, immunotherapy has significantly altered the therapeutic landscape for several solid cancers, becoming an integral part of standard therapeutic regimens and providing improved survival outcomes. Specifically, the inhibition of the PD‐1/PD‐L1 pathway has shown promise in increasing tumor immunogenicity and facilitating T‐cell mediated destruction of tumor cells [14]. The 2023 ESMO guidelines for CUP suggest that ICIs could be considered as a treatment option when clinicopathological features resemble those of known primaries where immunotherapy has proven effective [1]. However, the application of such immunotherapeutic approaches to the management of CUP remains less defined. Individual case reports have noted remarkable responses to immune checkpoint inhibitors (ICIs) in CUP [15, 16, 17, 18, 19, 20, 21, 22], and three recent clinical trials collectively reported an objective response rate (ORR) of approximately 20% among CUP patients treated with either monotherapy or dual immunotherapy, yielding a median PFS and OS of 2.5–4.1 and 3.8–15.9 months, respectively [23, 24, 25]. Although promising outcomes results have been reported, comprehensive real‐world data on immunotherapy in the treatment of CUP remain limited and could provide a valuable addition to clinical trials.

Even though immunotherapy revolutionary impact on the management of several cancer types, the therapeutic outcomes of immunotherapy alone in unfavorable CUP have not been entirely satisfactory. However, accumulating evidence supports the combination of ICIs with chemotherapy to treat cancer, suggesting a potential synergistic effect that could enhance overall treatment efficacy. Yet, the utilization of ICIs in real‐world settings is not fully understood, and the potential advantage of combining immunotherapy with conventional chemotherapy over chemotherapy alone for CUP remains unconfirmed, given the current scarcity of clinical trials specifically designed to rigorously evaluate this comparison. Given the rarity of this tumor and considering the enrollment duration of previous clinical trials [9, 26], initiating a large‐scale Phase III clinical trial to compare immunotherapy plus chemotherapy with chemotherapy alone is expected to require an extended period. Consequently, we conducted a multicenter, real‐world study to investigate the efficacy and safety of PD‐1/PD‐L1 inhibitors with chemotherapy for unfavorable CUP in the first‐line setting. Our results indicated that PD‐1/PD‐L1 inhibitors combined with chemotherapy significantly enhance efficacy compared to chemotherapy alone in this challenging patient population. The present study highlights the benefits of adding ICIs to chemotherapy in a real‐world setting as first‐line treatment and provides critical insights for the management of patients with unfavorable CUP.

## 2 Results

### 2.1 Patients and Treatments

**FIGURE 1:**
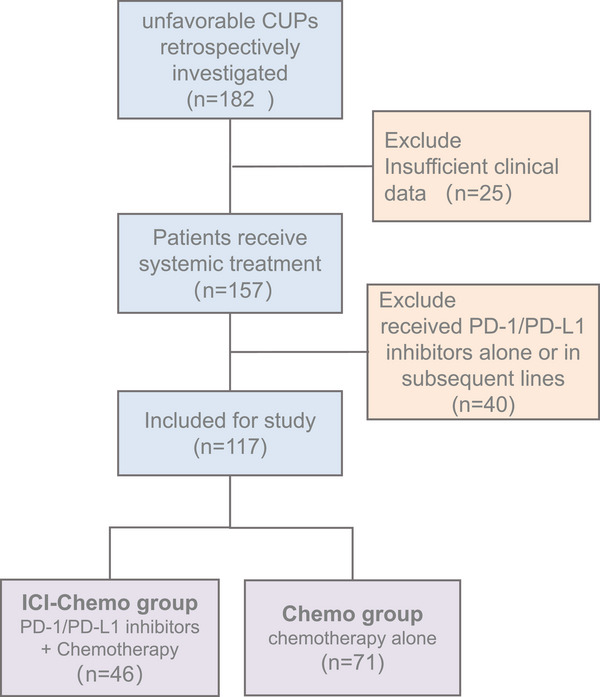
Flow chart of the study patients with cancer of unknown primary. CUP, cancer of unknown primary; ICI, immune checkpoint inhibitor; Chemo, chemotherapy.

Between January 2014 and December 2023, 117 patients with adequate clinical data and follow‐up information were included, with details of patient recruitment presented in Figure 1. In the entire cohort, 46 patients received PD‐1/PD‐L1 inhibitors plus chemotherapy (ICI‐Chemo group) and 71 patients received chemotherapy alone (Chemo group). Baseline disease characteristics of enrolled patients were well balanced between groups (Table 1), though the proportion of patients older than 60 years was greater in the ICI‐Chemo group than in the Chemo group (45.65% vs. 36.62%). The median age was 55.0 years, 58.97% were male, and 23.08% of them had a history of smoking. To identify the primary tumor, 101 patients (86.32%) underwent PET/CT, while the remaining patients were mainly diagnosed by computed tomography (CT), MRI, and endoscopy. At baseline, seven (15.22%) and 15 (32.61%) patients in the ICI‐Chemo group had liver and bone metastases, while the numbers were 12 (16.90%) and 24 (33.80%) in the Chemo group, respectively.

**TABLE 1:** Patient demographics.

	No. of patients (%)			
Characteristics	All patients (*n* = 117)	ICI‐Chemo (*n* = 46)	Chemo (*n* = 71)	*p*‐value
Age				
Median (range)	55 (21–80)	55 (21–80)	54 (27–71)	
≥60 years	47 (40.17)	21 (45.65)	26 (36.62)	0.435
Sex				
Female	48 (41.03)	17 (36.96)	31 (43.66)	0.565
Male	69 (58.97)	29 (63.04)	40 (56.34)	
ECOG performance status				
0	65 (55.56)	29 (63.04)	36 (50.70)	0.253
≥1	52 (44.44)	17 (36.96)	35 (49.30)	
Smoking history	27 (23.08)	10 (21.74)	17 (23.94)	0.826
Histology				
Adenocarcinoma	63 (53.85)	20 (43.48)	43 (60.56)	0.240
Squamous cell carcinoma	31 (26.50)	13 (28.26)	18 (25.35)	
Undifferentiated carcinoma	18 (15.38)	11(23.91)	7 (9.86)	
Other	5 (4.27)	2 (4.35)	3 (4.23)	
Site of metastasis				
Visceral disease^a^	81 (69.23)	32 (69.57)	49 (69.01)	1
Lymph node only	36 (30.77)	14 (30.43)	22 (30.99)	
Visceral metastasis site				
Peritoneal or omental implantation	30 (25.64)	8 (17.39)	22 (30.99)	0.13
Adrenal gland	7 (5.98)	4 (8.70)	3 (4.23)	0.431
Liver	19 (16.24)	7 (15.22)	12 (16.90)	1
Lung	17 (14.53)	5 (10.87)	12 (16.90)	0.43
Bone	39 (33.33)	15 (32.61)	24 (33.80)	1
Brain	3 (2.56)	2 (4.35)	1 (1.41)	0.561
Number of metastatic sites				
1	6 (5.13)	2 (4.35)	4 (5.63)	0.5397
2	24 (20.51)	7 (15.22)	17 (23.94)	
≥3	87 (74.36)	37 (80.43)	50 (70.42)	
Combination therapy				
TP‐based chemotherapy	68 (58.12)	27 (58.70)	41 (57.75)	1
Other	49 (41.88)	19 (41.30)	30 (42.25)	

Abbreviations: ECOG, Eastern Cooperative Oncology Group; TP, taxane‐platinum.

^a^ Lung, liver, bone, brain, or other non‐lymph node metastasis.

Among these patients, TP (taxane plus platinum)‐based chemotherapies were dominant in both groups, with 27 patients (58.70%) in the ICI‐Chemo group and 41 patients (57.75%) in the Chemo group. In the Chemo group, aside from TP‐based chemotherapy regimens, other commonly used regimens including XELOX (oxaliplatin + capecitabine) (9.86%) and FOLFOX (5‐fluorouracil + oxaliplatin + leucovorin) based chemotherapy (14.08%) were mainly used in patients with pathological diagnosis of adenocarcinoma. In the ICI‐Chemo group, patients were treated with ICIs approved by the FDA or China's National Medical Products Administration, such as sintilimab (26.09%), pembrolizumab (21.74%), and tislelizumab (17.39%).

### 2.2 Efficacy Profiling

The median follow‐up for the entire cohort was 28.1 months (95% CI: 17.57–37.63). In the ICI‐Chemo group, six patients (13.04%) completed 1 year of PD‐1/PD‐L1 inhibitors, and 10 patients (21.74%) received ICIs after progression. Treatment was ongoing in 10 patients (8.55%), with six patients in the ICI‐Chemo group and four patients in the Chemo group.

**FIGURE 2:**
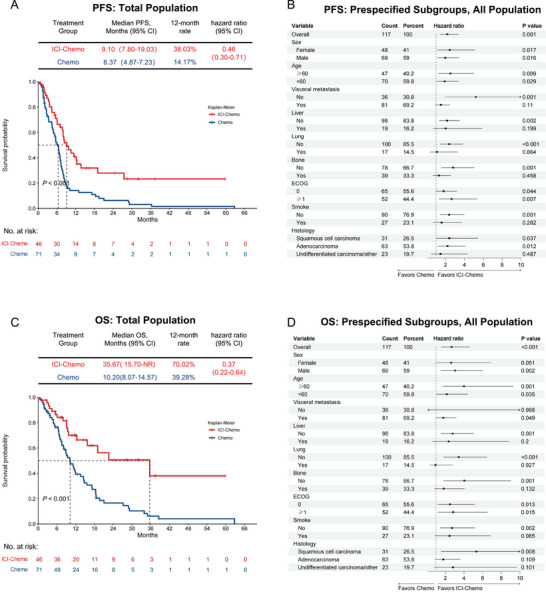
Progression‐free survival (PFS) and overall survival (OS): ICI‐Chemo Group versus Chemo Group. (A) Kaplan–Meier estimates of PFS in the total population and (B) an analysis of PFS in prespecified subgroups. (C) Kaplan–Meier estimates of OS in the total population and (D) an analysis of OS in prespecified subgroups.

The ICI‐Chemo group showed a benefit over the Chemo group in PFS. At the cut‐off date, 30 patients (65.22%) in the ICI‐Chemo group and 66 (78.95%) patients in the Chemo group had experienced progression or death. The median PFS was 9.10 months (95% CI: 7.80–19.03 months) in the ICI‐Chemo group, compared to 6.37 months (95% CI: 4.87–7.23 months) in the Chemo group (hazard ratio [HR] 0.46; 95% CI: 0.30–0.71; *p* < 0.001) (Figure 2A). At 12 months, the percentage of patients who were alive without disease progression was 38.03% in the ICI‐Chemo group as compared with 14.17% in the Chemo group (Figure 2A). The HRs for PFS favored the ICI‐Chemo group across several subgroups, especially in those without liver metastases, non‐smokers, and those with adenocarcinoma, consistently demonstrating a preference for ICI‐Chemo over chemotherapy alone (Figure 2B). At the data cut‐off, 17 (36.96%) of 46 individuals in the ICI‐Chemo group and 59 (83.10%) of 71 patients in the Chemo group had died. Median OS was 35.67 months (95% CI: 15.70–not reach [NR]) in the ICI‐Chemo group compared with 10.20 months (95% CI: 8.07–14.57) in the Chemo group (HR 0.37; 95% CI: 0.22–0.64; *p* < 0.001) (Figure 2C). The 12‐month OS rate was 70.02% in the ICI‐Chemo group as compared with 39.28% in the Chemo group (Figure 1C). The HRs for OS favored the ICI‐Chemo group in most prespecified subgroups, particularly in patients without liver metastasis, non‐smokers, and those with a performance status of ECOG 0 (Figure 2D).

To assess the impact of immunotherapy on PFS and OS, we performed univariate and multivariate analyses. These analyses revealed that the combination of ICIs with additional immunotherapy significantly improved PFS and OS (Table 2, Table S1). Additionally, both univariate and multivariate analyses demonstrated that the absence of bone metastasis was independently associated with enhanced OS in this patient cohort (Table 2). These findings indicated that the combination of ICIs and chemotherapy serves as independent prognostic factors for both PFS and OS in this patient population.

**TABLE 2:** Univariate and multivariate analyses for overall survival in 117 unfavorable CUP patients.

	Univariate analysis		Multivariate analysis	
Characteristic	HR (95% CI)	*p* value	HR (95% CI)	*p* value
Age				
<60	Reference			
≥60	1.39 (0.87, 2.20)	0.167		
Sex				
Female	Reference			
Male	1.33 (0.83, 2.12)	0.231		
ECOG				
≥1	Reference			
0	0.79 (0.50, 1.25)	0.321		
Smoke				
No	Reference			
Yes	1.15 (0.69, 1.92)	0.585		
Histology				
Adenocarcinoma	Reference			
Squamous cell carcinoma	0.78 (0.45, 1.33)	0.362		
Undifferentiated carcinoma/other	0.57 (0.30, 1.11)	0.100		
Visceral_metastasis				
No	Reference			
Yes	1.71 (1.01, 2.89)	0.045	1.13 (0.61, 2.07)	0.7
Liver				
No	Reference			
Yes	2.02 (1.07, 3.83)	0.030	1.91 (0.99, 3.72)	0.055
Lung				
No	Reference			
Yes	1.94 (1.10, 3.43)	0.022	1.54 (0.84, 2.82)	0.2
Bone				
No	Reference			
Yes	1.86 (1.16, 2.96)	0.010	1.82 (1.09, 3.03)	0.022
Number of metastatic sites				
≥3	Reference			
1	0.48 (0.15, 1.55)	0.219		
2	1.12 (0.65, 1.93)	0.675		
Treatment group				
Chemo	Reference		Reference	
ICI‐Chemo	0.37 (0.22, 0.64)	<0.001	0.37 (0.21, 0.64)	<0.001

Abbreviations: Chemo, chemotherapy; CI, confidence interval; ECOG, Eastern Cooperative Oncology Group Performance Status; HR, hazard ratio; ICI, immune checkpoint inhibitor,

The best overall response in each population is summarized in Table 3. Among all patients, four (3.42%) individuals had a confirmed complete response (CR) and 37 (31.62%) had a confirmed partial response (PR), yielding an ORR of 35.04% (95% CI: 26.39–43.69) for all populations. Three (6.52%) patients in the ICI‐Chemo group and only one (1.41%) in the Chemo group reached CR, while 22 (47.83%) in the ICI‐Chemo group and 15 (21.13%) in the Chemo group obtained PR as their best response. Progressive disease was the best overall response in five patients (10.87%) in the ICI‐Chemo group and 15 patients (21.13%) in the Chemo group. The ORR was 54.35% (95% CI: 39.96–68.74) in the ICI‐Chemo group and 22.53% (95% CI: 12.81–32.25) (*p* < 0.001) in the Chemo group. The disease control rate (DCR) showed no significant difference between the ICI‐Chemo group and the Chemo group (82.61% vs. 66.20%, *p* = 0.083).

**TABLE 3:** Summary of anti‐tumor activity.

Response	All patients (*n* = 117)	ICI‐Chemo (*n* = 46)	Chemo (*n* = 71)	*p* value
Objective response				
No. of patients	41	25	16	<0.001
Percentage of patients (95% CI)	35.04 (26.39–43.69)	54.35 (39.96–68.74)	22.53 (12.81–32.25)	
Best overall response, *N* (%)				
Complete response	4 (3.42)	3 (6.52)	1 (1.41)	
Partial response	37 (31.62)	22 (47.83)	15 (21.13)	
Stable disease	44 (37.61)	13 (28.26)	31 (43.66)	
Progressive disease	20 (17.09)	5 (10.87)	15 (21.13)	
No assessment	12 (10.26)	3 (6.52)	9 (12.68)	
Disease control^a^				
No. of patients	85	38	47	0.083
Percentage of patients (95% CI)	72.65 (64.57–80.73)	82.61 (71.66–93.56)	66.20 (55.20–77.20)	

^a^ Disease control was defined as complete response, partial response, or stable disease at 6 weeks with no progression.

**FIGURE 3:**
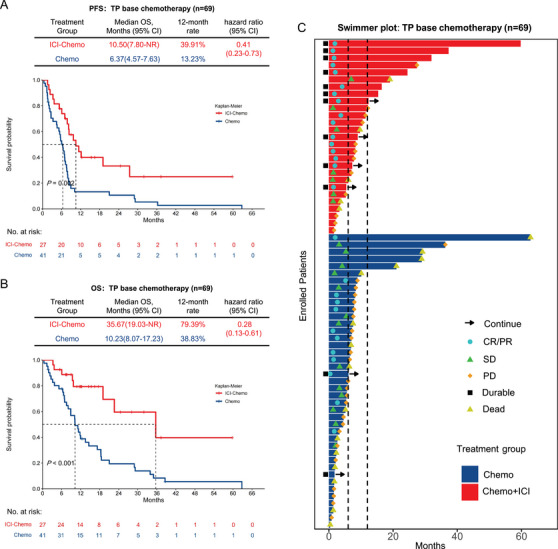
Survival outcomes for the population who underwent TP‐based chemotherapy regimens: (A) Progression‐free survival and (B) overall survival for the ICI‐Chemo group and the Chemo group. (C) Swimmer plot of progression‐free survival. Each bar represents one subject in the study. Arrows indicate patients still on treatment.

To further elucidate the advantage of adding ICIs to chemotherapy, a TP‐based regimen‐specific analysis was conducted since the TP was dominant. In this population, baseline demographics were balanced between the ICI‐Chemo group and the Chemo group (Table S2). In the population receiving TP‐based regimens, the ICI‐Chemo group exhibited a significant improvement over the Chemo group in median PFS (10.50 months vs. 6.37 months; HR 0.41; 95% CI: 0.23–0.73; *p* = 0.002) (Figure 3A) and OS (35.67 months vs. 10.23 months; HR 0.28; 95% CI: 0.13–0.61; *p* < 0.001) (Figure 3B). The ORR was higher in the ICI‐Chemo group (59.26%, 95% CI: 40.73–77.79) than the Chemo group (29.27%, 95% CI: 15.34–43.20; *p* = 0.027) (Table S3). Of note, at the time of data cut‐off, 10 patients (37.04%) in the ICI‐Chemo group had an ongoing response lasting >12 months, while only five patients (12.20%) in the Chemo group exhibited ongoing responses lasting >12 months (Figure 3C). However, among patients who did not receive the TP regimen but received other regimens, the ICI‐Chemo group showed a longer PFS (7.97 months vs. 6.5 months, *p* = 0.115) and OS (12.37 months vs. 9.03 months, *p* = 0.134) than the Chemo group, but statistical significance was not reached.

Among the 17 patients (14.5%) with available PD‐L1 testing results, 12 in the ICI‐Chemo group showed that seven exhibited a combined positive score (CPS) ≥ 10 and five had a CPS < 10, whereas all five patients in the Chemo group demonstrated a CPS < 10. In the population with PD‐L1 CPS < 10, four individuals in the Chemo group had disease progression or death, and none maintained ongoing response for more than 6 months, while two out of five patients in the ICI‐Chemo group had progressed or died, and four maintained response >6 months (Figure S1). Notably, in the ICI‐Chemo group, four out of seven patients with PD‐L1 CPS ≥ 10 sustained response for over 12 months, while none of those with PD‐L1 CPS < 10 exhibited ongoing responses lasting more than 12 months (Figure S1).

Among these patients, 24 (20.51%) with 25 samples performed next‐generation sequencing. The Oncoplot illustrated the mutations and distribution of genetic variants across these patients (Figure S2). The most frequent mutations identified were *TP53* alterations (52%), *PIK3CA* alterations (20%), and *KRAS* mutations (16%). None of the samples showed microsatellite instability (MSI). The median tumor mutation burden (TMB) was 3.84 mutations per megabase (Muts/Mb). Patients with TMB above the median who received immunotherapy showed better outcomes, with two out of seven patients achieving PFS more than 2 years (Figure S3).

### 2.3 Safety Profiling

The incidences of any adverse events (AEs) and Grade 3–4 AEs for all patients are presented in Table 4. The ICI‐Chemo group reported AEs in 97.83% of patients, compared to 90.14% in the Chemo‐only group. Grade 3–4 AEs were observed in 23.91% of the ICI‐Chemo group and 39.44% of the Chemo group. In both groups, the most prevalent AEs of any grade included anemia, leukopenia, and neutropenia, while the most frequent grades 3–4 AEs were anemia and neutropenia. No treatment‐related death was reported. AEs observed were in line with the established safety profile of ICIs. Immune‐related AEs were documented in 15 (32.61%) patients, predominantly as Grade 1 or 2 events. The most frequent immune‐mediated AEs in the ICI‐Chemo group included hypothyroidism (17.39%), pruritus (10.87%), and rash (6.52%). Additionally, one patient experienced Grade 3 immune‐related pneumonia, which required discontinuation of treatment.

**TABLE 4:** Treatment‐related adverse event.

	ICI‐Chemo (*n* = 46), No. (%)		Chemo (*n* = 71), No. (%)	
	Any grade	Grade 3 or 4	Any grade	Grade 3 or 4
Any AE	45 (97.83)	11 (23.91)	64 (90.14)	28 (39.44)
Leukopenia	23 (50.00)	3 (6.52)	37 (52.11)	8 (11.27)
Neutropenia	21 (45.65)	3 (6.52)	31 (43.66)	12 (16.90)
Febrile neutropenia	2 (4.35)	2 (4.35)	2 (2.82)	2 (2.82)
Thrombocytopenia	8 (17.39)	5 (10.87)	10 (14.08)	3 (4.23)
Anemia	37 (80.43)	6 (13.04)	52 (73.24)	9 (12.68)
Serum creatinine increased	8 (17.39)	0	10 (14.08)	0
Elevated transaminases	17 (36.96)	0	31 (43.66)	3 (4.23)
Fatigue	20 (43.48)	0	4 (5.63)	0
Dyspepsia	22 (47.83)	0	7 (9.86)	0
Nausea	19 (41.30)	0	12 (16.90)	0
Vomiting	14 (30.43)	0	11 (15.49)	0
Diarrhea	3 (6.52)	0	4 (5.63)	0
Constipation	13 (28.26)	0	2 (2.82)	0
Peripheral neuropathy	6 (13.04)	0	6 (8.45)	1 (1.41)
Immune‐related AEs				
Any irAE	15 (32.61)	1 (2.17)	0	0
Rash	3 (6.52)	0	0	0
Pruritus	5 (10.87)	0	0	0
Hypothyroidism	8 (17.39)	0	0	0
Hyperthyroidism	1 (2.17)	0	0	0
Adrenal_insufficiency	1 (2.17)	0	0	0
Hepatitis	2 (4.35)	0	0	0
Pneumonia	2 (4.35)	1 (2.17)	0	0

## 3 Discussion

CUP is a highly heterogeneous and life‐threatening malignancy with limited therapeutic options. In this present real‐world study, we investigated the efficacy and safety of PD‐1 inhibitors plus chemotherapy compared to chemotherapy alone as a first‐line therapy for patients with unfavorable CUP. To our knowledge, this study is the first and largest multi‐institutional comparison of PD‐1/PD‐L1 inhibitors plus chemotherapy with chemotherapy alone for this patient population. Given the scarcity of unfavorable CUP and the limited availability of large‐scale prospective data, this study offers crucial clinical insights into the treatment of unfavorable CUP with PD‐1/PD‐L1 inhibitors.

CUP has historically been considered a single entity, which has made it challenging to conduct phase III trials to explore new therapeutic alternatives. A meta‐analysis of five original studies, which included 454 patients receiving site‐specific therapy and 660 receiving empiric therapy, found no significant improvement in PFS or OS with site‐specific therapy compared to empirical therapy [27]. Although historical empiric chemotherapy for CUP patients yields an ORR of 10%–40%, its survival advantage remains limited, with a PFS of 3–6 months and an OS of 6–13 months [3, 4, 5, 6, 7, 8, 9, 10, 11]. In our study, the PFS and OS of unfavorable CUP receiving chemotherapy alone were 6.37 months and 10.20, respectively, consistent with prior studies. These outcomes underscore the challenges of achieving significant improvements in results with conventional chemotherapy alone. The limited efficacy observed with chemotherapy alone in our study highlights the urgent unmet medical need for novel therapeutic approaches, particularly in patients with poor prognoses.

The introduction of immunotherapy, particularly PD‐1/PD‐L1 inhibitors, has reshaped the treatment landscape for various malignancies, including unfavorable CUP. In 2019, a phase 2 clinical trial known as the NivoCUP‐2 trial was conducted to assess the efficacy of nivolumab for CUP, which enrolled 45 previously treated patients and 11 previously untreated patients [24]. The trial demonstrated an improvement in the PFS rate, irrespective of treatment history [24]. Additionally, the treated group showed an improvement in OS rate, whereas the untreated group did not achieve this outcome. The results from the NivoCUP‐2 trial contributed to the approval of nivolumab as a first‐line treatment, but the response rate was only 18.2%, indicating that its impact on tumor shrinkage was limited [24]. Another phase 2 clinical trial investigated the combination of nivolumab and ipilimumab in previously treated unfavorable CUP patients, and despite this combination therapy, the overall response rate remained modest, at just 16% [23]. Thus, while immunotherapy has shown promise in the management of unfavorable CUP, further optimization and combination strategies are warranted to enhance treatment efficacy and improve patient outcomes.

PD‐1/PD‐L1 inhibitors combined with chemotherapy prolong survival in several entities, regardless of PD‐L1 expression level. The results of this study were consistent with those from the randomized studies of chemotherapy plus immunotherapy against chemotherapy alone in other tumors. By comparison, the survival benefit seen in this study highlights the potential of PD‐1/PD‐L1 inhibitors to confer a better outcome in such patients. In our patient cohort, the combination of anti‐PD‐1 therapy and chemotherapy reduced the risk of disease progression or death by 54% compared to chemotherapy alone. The introduction of immunotherapy also yielded benefits in tumor response, as indicated by enhanced ORR and DOR. Nevertheless, given the heterogeneity of CUP and the lack of validated predictive biomarkers, optimal patient selection remains elusive.

Several predictive biomarkers indicative of a potential response to ICIs, such as high PD‐L1 expression, MSI, and TMB, may be relevant for a significant subset of CUP cases. Prior research has revealed that 28% of CUP patients exhibit one or more biomarkers predictive of response to ICIs^28^. Genomic analyses of CUP have identified that approximately 10%–20% of instances possess elevated TMB levels, which vary based on the applied TMB threshold [23, 29, 30, 31, 32], and CUP patients with low TMB levels tend to have unfavorable outcomes when treated with ICIs [23, 28]. However, to determine the predictive value of these biomarkers for ICIs plus chemotherapy in CUP, prospective data involving larger cohorts of treatment efficacy and biomarker reliability are needed.

The limitation of this study is its retrospective nature, which introduces inherent heterogeneity and potential biases. The study is further constrained by insufficient data on CPS status, rendering the conclusions on the impact of PD‐L1 on immunotherapy outcomes in CUP preliminary and requiring larger scale studies for validation. The inclusion of more biomarker information (such as tumor mutational burden, tumor microenvironment indicators, etc.) is needed in the future. Due to the retrospective nature, some selection bias may be introduced. However, we reviewed the baseline characteristics and confirmed both treatment groups were comparably balanced, which helps to alleviate concerns about potential bias. However, the study has several advantages, including clinical data from multiple medical centers in a real‐world setting, and it provides the largest cohort of patients with unfavorable CUP to compare the efficacy and safety of first‐line PD‐1/PD‐L1 inhibitors plus chemotherapy versus chemotherapy alone, providing valuable supplementary insights to clinical trials. Nevertheless, further prospective clinical trials involving an even larger cohort of patients with unfavorable CUP are essential to validate these findings. Additionally, efforts should be made to identify predictive biomarkers that can accurately select unfavorable CUP patients who are most likely to benefit from immunotherapy‐based approaches.

In conclusion, our study demonstrated that PD‐1/PD‐L1 inhibitors plus chemotherapy significantly improved the efficacy compared to chemotherapy alone with manageable toxicity, and this approach could be the preferred first‐line treatment for patients with unfavorable CUP.

## 4 Methods

### 4.1 Study Design and Population

This multicenter, retrospective, real‐world study included patients with unfavorable CUP across four medical centers in China: Sun Yat‐sen University Cancer Center [SYSUCC], the First Affiliated Hospital of Sun Yat‐sen University, Central Hospital of Guangdong Nongken, and First People's Hospital of Zhaoqing City. These institutions provide a representative sample of patients from diverse backgrounds. Each center meticulously reviewed its medical records to identify and include as many eligible cases as possible. The efficacy and safety of first‐line treatment were investigated between the ICI‐Chemo group and the Chemo group. Eligible patients were ≥18 years old, met clinical and histologic criteria for unfavorable CUP, and received at least one dose of first‐line treatment. To not affect the OS of the Chemo group, patients who received immunotherapy for subsequent treatment were excluded from this group. All cases had comprehensive clinical documentation, and primary lesions were not detected through various diagnostic tools such as X‐rays, CT, MRI, PET/CT, and endoscopy. Finally, all initially screened patients were reviewed by SYSUCC oncologists and pathologists to select CUP patients. Pathological analysis was conducted to exclude malignant melanoma, sarcoma, malignant lymphoma, and neuroendocrine tumor. Patient classification into favorable or unfavorable groups followed the ESMO guidelines [1]; individuals in the favorable group were excluded. The study protocol was assessed and approved by the ethical committees of each respective institution: SYSUCC (approval number B2023‐684‐01), the First Affiliated Hospital of Sun Yat‐sen University (approval number [2024]497), Central Hospital of Guangdong Nongken (approval number 24134), and First People's Hospital of Zhaoqing City (approval number B2024‐07‐01). The committee waived the necessity for individual informed consent because of the low‐risk nature associated with this study. This research complied with the principles outlined in the Declaration of Helsinki.

### 4.2 Data Collection and Definitions

The data were obtained from patient medical records, including demographic information, tumor characteristics, treatment details, laboratory results, and image scans. The PD‐1 antibody was prescribed by experienced oncologists and administered following instructions. Treatment decisions between ICIs plus chemotherapy or chemotherapy alone were made by a multidisciplinary team (MDT) or the attending clinicians, taking into account factors such as patient performance status, underlying comorbidities, and personal preferences. Despite the lack of specific approval for PD‐L1 inhibitors in CUP, their use was guided by previous clinical trials and ESMO guidelines. The decision‐making process was informed by the demonstrated benefits of immunotherapy in other cancers and was aligned with patient preferences, ensuring that it was a patient‐derived decision made with full informed consent.

Treatment‐related AEs were assessed according to the National Cancer Institute Common Terminology Criteria for Adverse Events version 5.0 (CTCAE 5.0). The relative frequency of each AE related to chemotherapy or immunotherapy was calculated as the proportion of all toxicity‐evaluable cycles with the event. The cohort for safety analysis comprised all participants who were administered at least one dose of the prescribed treatment regimen. This approach ensures a comprehensive assessment of treatment tolerability and safety across the diverse therapies administered in the study.

Disease assessment was conducted in accordance with the RECIST version 1.1 to evaluate treatment responses. Survival was assessed from the start of therapy until death. Additionally, the DCR, ORR, PFS, and OS were evaluated. PFS was defined as the time from the initiation of treatment to the progression of disease (PD) or death. OS was measured from the start of treatment until death from any cause. The DCR was measured as the percentage of patients who reached a CR, PR, or stable disease (SD) based on the RECIST 1.1. The ORR was assessed by the proportion of patients who attained a CR or a PR.

PD‐L1 expression was evaluated on both tumor cells and mononuclear inflammatory cells within tumor nests through immunohistochemical analysis performed by the pathology department at our institution using the Merck 22C3 antibody. The CPS for PD‐L1 was determined by counting PD‐L1‐staining cells (including tumor cells, lymphocytes, and macrophages), dividing by the total tumor cell count, and then multiplying by 100 [33]. PD‐L1 positivity was defined as CPS ≥ 1.

### 4.3 Statistical Analysis

Statistical analyses and data visualization were conducted using R version 4.2.2 (The R Project for Statistical Computing, www.r‐project.org) and SPSS 25.0 (IBM, Armonk, NY, USA). Descriptive statistics, including percentages and medians, were used to summarize patient characteristics, disease features, diagnostic work‐up, and treatments administered. Data were analyzed up to a cut‐off date of March 8, 2024. Time‐to‐event outcomes for each treatment cohort were estimated using the Kaplan–Meier method, and comparisons of OS and PFS between treatment groups were performed using the stratified log‐rank test. Cox proportional hazard models were employed to calculate hazard ratios with 95% CIs. Univariable and multivariable Cox models were utilized to investigate the prognostic value of covariates. The incidence and severity of AEs were assessed within the safety analysis set. A two‐sided *p* value < 0.05 was considered statistically significant.

## Author Contributions

Y.S. has complete access to all the study data and takes responsibility for the integrity and accuracy of the data and its analysis. Y.S., Y.C., and R.H. designed the study. R.H., M.C., S.L., Y.H., Z.Z., D.S., T.G., A.H., and R.C. collected the data. H.L., R.H., and S.L. analyzed and interpreted the data. R.H. and H.L. drafted the manuscript. Y.S. and Y.C. supervised and provided critical revisions to the manuscript for important intellectual content. Y.S., C.X., Q.Z., and X.A. provided administrative, technical, and material support. All authors reviewed the manuscript and offered final approval of the manuscript.

## Ethics Statement

The clinical data were collected with approval from the Institutional Review Board of the Sun Yat‐sen University Cancer Center. The study protocol received approval from the ethical committees of each respective institution: SYSUCC (approval number B2023‐684‐01), the First Affiliated Hospital of Sun Yat‐sen University (approval number [2024]497), Central Hospital of Guangdong Nongken (approval number 24134), and First People's Hospital of Zhaoqing City (approval number B2024‐07‐01). The study was conducted following the principles of the Declaration of Helsinki. All procedures were carried out in compliance with relevant guidelines and regulations.

## Consent

The requirement for informed consent was waived by the ethical committee of the Sun Yat‐sen University Cancer Center, as the study involved a retrospective analysis of clinical data with no ethical concerns related to human biology.

## Conflicts of Interest

The authors declare no conflicts of interest.

## Supporting information



Supporting Information

## Data Availability

The datasets generated in this study are available from the corresponding author upon reasonable request. The key raw data have been made available on the Research Data Deposit public platform (https://www.researchdata.org.cn/, RDDA2024578722).
