# Supplementary material for: PD‐1/PD‐L1 Inhibitors Plus Chemotherapy Versus Chemotherapy Alone as First‐Line Therapy for Patients With Unfavorable Cancer of Unknown Primary: A Multicenter, Retrospective Cohort Study
Source: MedComm (2020). 2025 Mar 6;6(3):e70124. doi: 10.1002/mco2.70124 (PMC11885889; doi:10.1002/mco2.70124)
Supplement: Supplementary file 1 — Supporting Information [file MCO2-6-e70124-s001.pdf]

Figure S1

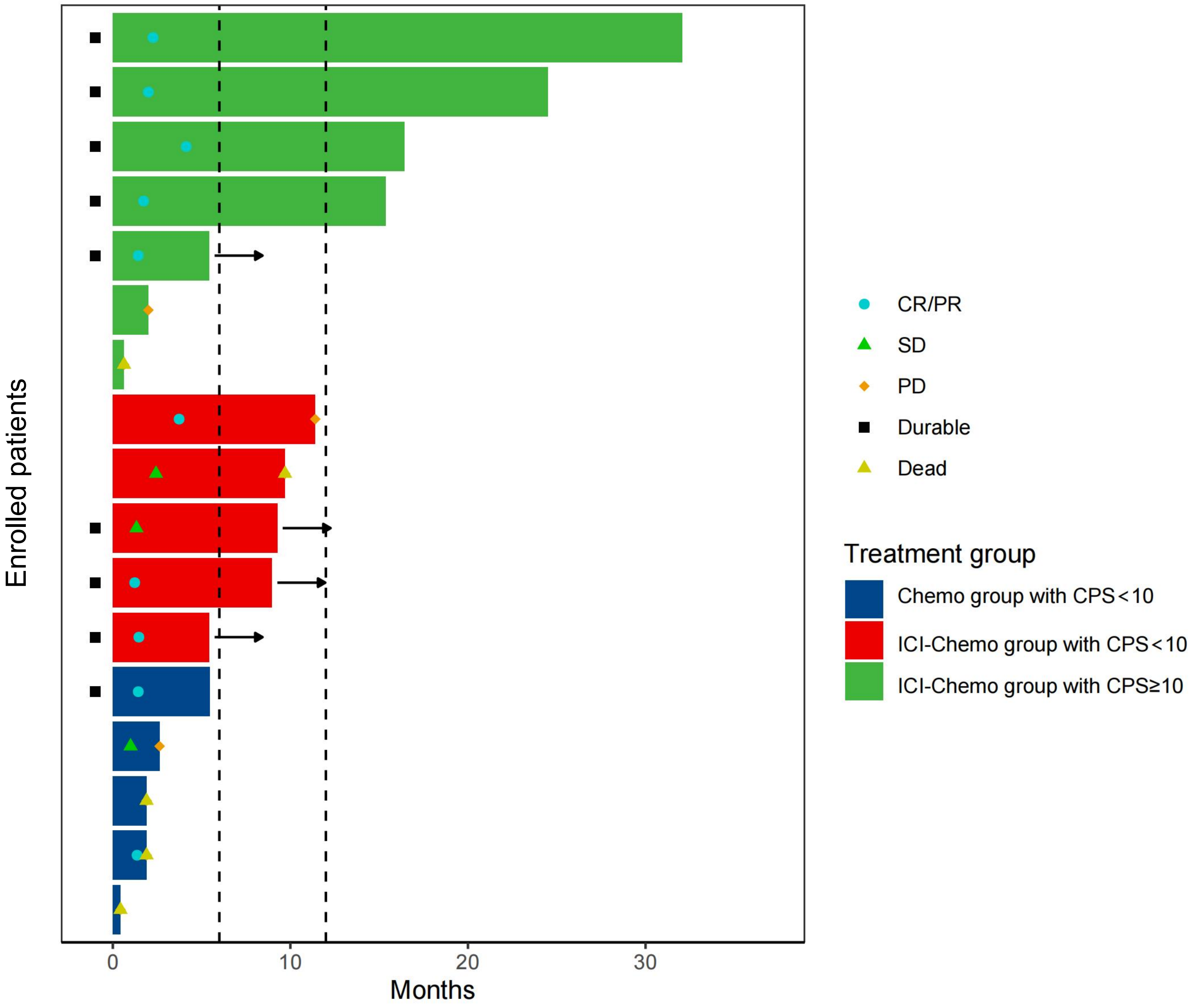

Figure S1. Swimmer plot of progression-free survival in patients with different PD-L1 expression levels. Arrows indicate patients still on treatment.

Figure S2

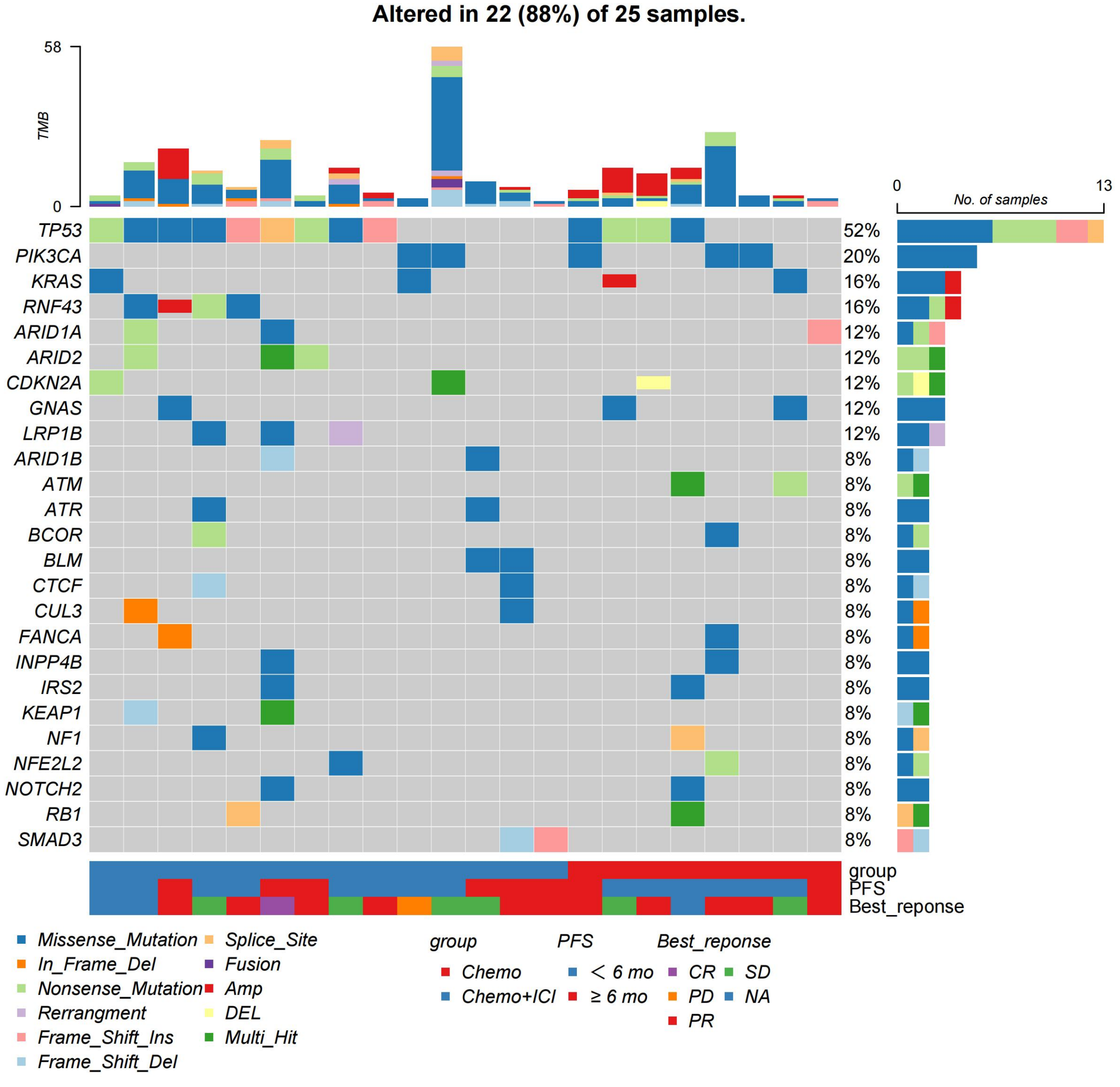

Figure S2. The landscape of frequently altered genes in patients with available genomic profiling data.

Figure S3

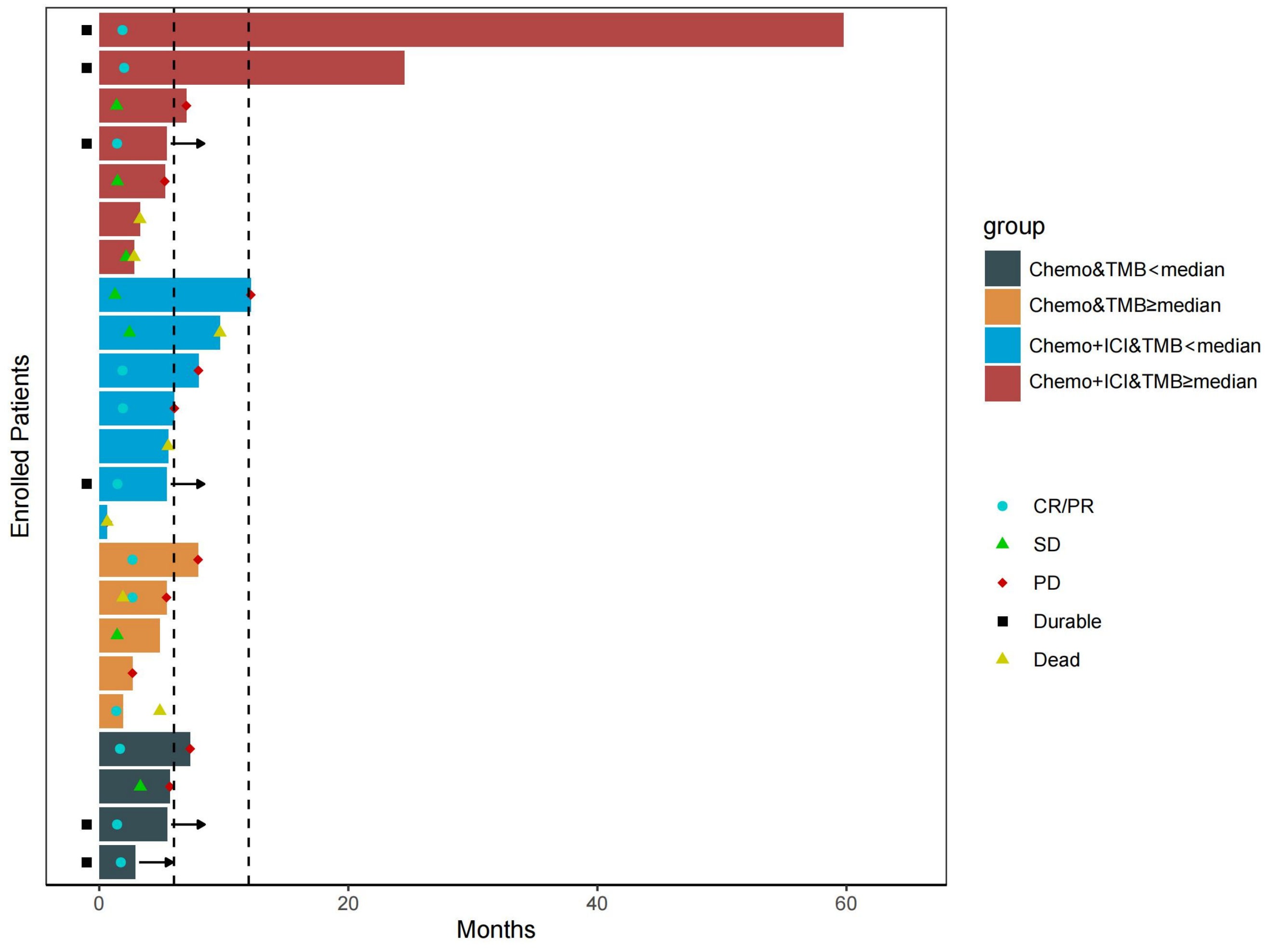

Figure S3. Swimmer plot of progression-free survival in patients with available tumor mutation burden data.

Table S1. Univariate and multivariate analyses for progression-free survival in 117 unfavorable CUP

Characteristic	Univariate analysis		Multivariate analysis	
	HR(95% CI)	*P* value	HR(95% CI)	*P* value
Age				
＜60	Reference			
≥60	1.04(0.69, 1.57)	0.865		
Sex				
Female	Reference			
Male	1.19(0.79, 1.80)	0.410		
ECOG				
≥1	Reference			
0	0.96(0.64, 1.44)	0.854		
Smoke				
No	Reference			
Yes	1.28(0.80, 2.03)	0.298		
Histology				
Adenocarcinoma	Reference			
Squamous cell carcinoma	0.93(0.58, 1.48)	0.752	0.95(0.59, 1.53)	0.800
Undifferentiated carcinoma/other	0.59(0.32, 1.06)	0.076	0.62(0.34, 1.13)	0.120
Visceral_metastasis				
No	Reference			
Yes	1.45(0.91, 2.31)	0.115		
Liver				
No	Reference			
Yes	1.72(0.99, 2.97)	0.054	1.70(0.97, 2.97)	0.062
Lung				
No	Reference			
Yes	1.21(0.70, 2.07)	0.494		
Bone				
No	Reference			
Yes	1.54(1.02, 2.34)	0.041	1.49(0.98, 2.29)	0.065
Number of metastatic sites				
≥3	Reference			
1	0.90(0.36, 2.25)	0.824		
2	1.22(0.76, 1.98)	0.413		
Treatment group				
Chemo	Reference			
ICI-Chemo	0.46(0.30, 0.71)	<0.001	0.48(0.31, 0.76)	0.001

Abbreviations: HR, Hazard Ratio; CI, Confidence Interval; ECOG, Eastern Cooperative Oncology Group Performance Status; ICI, immune checkpoint inhibitor ; Chemo, chemotherapy.

patients.

Table S2. Patient demographics in patients who received TP-Based chemotherapy

Characteristics	No. of patients (%) All patients (n=68)	ICI-Chemo (n = 27	Chemo (n = 41)	*P*-value
Age				
Median (range)	54(21-75)	54(21-75)	54(27-71)	0.9863
<65 years	39 (57.35)	14 (51.85)	25 (60.98)	0.6215
Sex				
Female	28 (41.18)	11 (40.74)	17 (41.46)	1
Male	40 (58.82)	16 (59.26)	24 (58.54)	
ECOG performance status				
0	38 (55.88)	17 (62.96)	21 (51.22)	0.4549
≥1	30 (44.12)	10 (37.04)	20 (48.78)	
Smoking history	23 (33.82)	8 (29.63)	15 (36.59)	0.6087
Histology				
Adenocarcinoma	3 (4.41)	0 (0.00)	3 (7.32)	0.1584
Squamous cell carcinoma	28 (41.18)	12 (44.44)	16 (39.02)	
Undifferentiated carcinoma	13 (19.12)	8 (29.63)	5 (12.20)	
Other	4 (5.88)	2 (7.41)	2 (4.88)	
Site of metastasis				
Visceral disease[a]	39 (57.35)	17 (62.96)	22 (53.66)	0.4664
Lymph node only	29 (42.65)	10 (37.04)	19 (46.34)	
Visceral metastasis site				
Peritoneal or omental implantation	7 (10.29)	1 (3.70)	6 (14.63)	0.2301
Adrenal gland	4 (5.88)	2 (7.41)	2 (4.88)	1
Liver	7 (10.29)	4 (14.81)	3 (7.32)	0.4231
Lung	8 (11.76)	3 (11.11)	5 (12.20)	1
Bone	25 (36.76)	11 (40.74)	14 (34.15)	0.6152
Brain	2 (2.94)	1 (3.70)	1 (2.44)	1

[a]Lung, liver, bone, brain, or other non-lymph node metastasis.

Abbreviations: ECOG, Eastern Cooperative Oncology Group; TP, taxane-platinum.

Table S3. Summary of antitumour activity for patients who received TP-Based chemotherapy

Response	All patients (n=68)	ICI-Chemo (n = 27	Chemo (n = 41)	*P* value
Objective response				
No. of patients	28	16	12	0.027
Percentage of patients (95% CI)	41.18 (29.48-52.88)	59.26 (40.73-77.79)	29.27 (15.34-43.20)	
Best overall response, N (%)				
Complete response	3(4.41)	2(7.41)	1(2.44)	
Partial response	25(36.76)	14(51.85)	11(26.83)	
Stable disease	20(29.41)	7(25.93)	13(31.71)	
Progressive disease	13(19.12)	3(11.11)	10(24.39)	
No assessment	7(10.29)	1(3.70)	6(14.63)	
Disease control[b]				
No. of patients	48	23	25	0.061
Percentage of patients (95% CI)	70.59 (59.76-81.42)	85.19 (71.79-98.59)	60.98 (46.05-75.91)	

[b]Disease control was defined as complete response, partial response, or stable disease at 6 weeks with no progression.
